# Radiotherapy Reprograms Intermediate Monocytes Into Proinflammatory Drivers of Systemic Inflammation in Radiation‐Induced Heart Disease

**DOI:** 10.1155/crp/1117746

**Published:** 2026-04-17

**Authors:** Jinchen He, Dejun Kong, Chengwei Zhang, Qiuhong Chen, Hong Yang, Yuyuan Wang, Tianqi Wu, Qi Wu

**Affiliations:** ^1^ Department of Cardiology, The Second Affiliated Hospital of Chengdu Medical College, Nuclear Industry 416 Hospital, Chengdu, Sichuan, 610051, China, scu.edu.cn; ^2^ Irradiation Preservation and Effect Key Laboratory of Sichuan Province, Chengdu, Sichuan, 610051, China, scu.edu.cn; ^3^ Department of Oncology, The Second Affiliated Hospital of Chengdu Medical College, Nuclear Industry 416 Hospital, Chengdu, Sichuan, 610051, China, scu.edu.cn

**Keywords:** flow cytometry, inflammatory remodeling, intermediate monocytes, pseudotime trajectory, RIHD, single-cell RNA sequencing

## Abstract

Radiation‐induced heart disease (RIHD) is a serious complication of thoracic radiotherapy, and its pathogenesis involves systemic immune alterations. To elucidate these mechanisms, we profiled peripheral blood mononuclear cells (PBMCs) from patients before and after thoracic radiotherapy using single‐cell RNA sequencing (scRNA‐seq), with key findings validated via multiparameter flow cytometry and ELISA assays. Our analysis revealed that radiotherapy markedly reshaped the immune composition, expanding innate myeloid cells (monocytes and neutrophils) and reducing lymphocytes (T and NK cells); these compositional shifts were independently confirmed by flow cytometric analysis. Cell–cell communication networks showed that postradiotherapy monocytes evolved into central signaling hubs, exhibiting heightened proinflammatory ligand–receptor interactions. Consistent with this, monocytes showed broad transcriptional reprogramming with upregulation of canonical inflammatory pathways (IL‐6/STAT3, TNF/NF‐κB) and metabolic regulators (mTORC1, glycolysis). ELISA assays corroborated these transcriptomic signatures, demonstrating significantly elevated plasma levels of IL‐6 and TNF‐α post‐treatment. Notably, scRNA‐seq identified a selective expansion of the highly plastic intermediate (CD14++CD16+) monocyte subset, a specific population shift further verified by flow cytometry. These intermediate monocytes exhibited an immature, progenitor‐like profile and were enriched at the origin of a Monocle3 pseudotime trajectory. Trajectory analysis indicated they differentiate into mature classical monocytes that upregulate proinflammatory effector genes such as S100A8, S100A9, and S100A12. Furthermore, pseudotime gene clustering revealed a functional bifurcation in monocyte behavior: one module drove inflammatory activation, while a second module simultaneously engaged oxidative stress responses and antioxidant defenses (e.g., glutathione metabolism). In summary, by integrating single‐cell transcriptomics with experimental validation, we demonstrate that radiotherapy drives a systemic immune shift characterized by intermediate monocyte expansion and bifurcated programs of inflammation and stress adaptation. Intermediate monocytes emerge as key drivers of postradiotherapy inflammation, offering a potential cellular biomarker of RIHD risk and a target for immunomodulatory interventions to mitigate cardiovascular injury in cancer survivors.

## 1. Introduction

Radiation‐induced heart disease (RIHD) is a significant nonmalignant complication arising from thoracic radiotherapy [1]. Traditionally, the pathogenesis of RIHD has been attributed to direct damage to cardiac tissues, including endothelial dysfunction and myocardial fibrosis [2–4]. However, emerging evidence suggests that systemic immune responses, particularly involving circulating immune cells, play a crucial role in the development and progression of RIHD [5, 6].

Monocytes, as pivotal components of the innate immune system, exhibit remarkable plasticity and responsiveness to environmental stimuli [7, 8]. They are broadly categorized into classical (CD14^++^CD16^−^), intermediate (CD14^++^CD16^+^), and nonclassical (CD14^+^CD16^++^) subsets, each with distinct phenotypic and functional characteristics [9, 10]. Among these, intermediate monocytes are noted for their high plasticity and proinflammatory potential, positioning them as key players in inflammatory responses.

Recent studies have highlighted the role of monocytes in mediating inflammatory processes following radiation exposure. For instance, radiation has been shown to induce the recruitment and activation of monocytes, leading to their differentiation into proinflammatory macrophages that contribute to tissue damage [11, 12]. Furthermore, single‐cell RNA sequencing analyses have revealed significant heterogeneity within monocyte populations, underscoring the complexity of their responses to radiation‐induced stress [13, 14].

Despite these insights, the specific contributions of intermediate monocytes to systemic immune remodeling and their role in the pathogenesis of RIHD remain poorly understood. Given their inherent plasticity and capacity for rapid response to inflammatory cues, intermediate monocytes may serve as critical mediators linking radiation exposure to subsequent cardiac injury.

In this study, we employed single‐cell transcriptomic profiling of peripheral blood mononuclear cells (PBMCs) collected from patients before and after thoracic radiotherapy. By integrating analytical tools such as CellChat, GSVA, CytoTRACE, and Monocle3, we aimed to delineate the alterations in immune cell composition and intercellular communication networks postradiotherapy, characterize the transcriptional and metabolic reprogramming of monocytes—with a focus on intermediate subsets—and trace the differentiation trajectories of monocyte subsets in response to radiation exposure. Our findings provide novel insights into the mechanisms by which intermediate monocytes contribute to systemic inflammation and cardiac injury following radiotherapy, offering potential avenues for therapeutic intervention in RIHD.

## 2. Materials and Methods

### 2.1. Patient Samples and Clinical Data Collection

Peripheral blood samples were collected from patients diagnosed with thoracic malignancies who received radiotherapy at The Second Affiliated Hospital of Chengdu Medical College, China National Nuclear Corporation 416 Hospital between 2023 and 2024. A total of six patients were included based on the following prespecified inclusion criteria: (1) age > 18 years; (2) pathologically confirmed thoracic malignancy; (3) planned curative‐intent thoracic radiotherapy with a mean heart dose (MHD) of ≥ 30 Gy; and (4) confirmed clinical diagnosis of RIHD during postradiotherapy follow‐up. Exclusion criteria included (1) active systemic infections or autoimmune diseases at baseline; (2) prior history of thoracic irradiation; or (3) documented pre‐existing cardiomyopathy or heart failure (LVEF < 50%) prior to cancer treatment.

Sample timing: to assess the acute and subacute effects of radiation, matched peripheral blood samples were collected at two rigid time points: (1) baseline (pre‐RT): collected within 24 h prior to the first fraction of radiotherapy; and (2) postradiotherapy (post‐RT): collected within 24 h following the final fraction of the radiotherapy course. The pre‐RT sample served as the within‐patient self‐control.

The mean age was 59.2 years, and 66.7% were male. The most common malignancy was lung cancer. Baseline cardiac function and inflammatory markers were within normal range prior to radiotherapy (Table 1). All patients provided written informed consent, and the study protocol was approved by the institutional ethics committee.

**TABLE 1:** Baseline characteristics of the patients undergoing thoracic radiotherapy (*n* = 6).

Clinical parameter	Value
Age (years), mean ± SD	59.2 ± 6.3
Sex (Male/Female)	4/2
Tumor type	Lung cancer (*n* = 4), Esophageal cancer (*n* = 2)
Planned total radiation dose (Gy)	60.0 ± 6.0
Heart dose (Dmean, Gy)	32.5 ± 3.2
LVEF (%), preradiotherapy	64.0 ± 3.5
BNP (pg/mL), preradiotherapy	85.4 ± 22.1
Baseline WBC count (× 10^9^/L)	6.1 ± 1.4
C‐reactive protein (CRP, mg/L)	3.7 ± 1.9
Comorbidities	Hypertension (*n* = 3), Diabetes (*n* = 2)
Smoking status (current/former/never)	4/0/2
Autoimmune diseases	None
Infectious diseases at baseline	None

### 2.2. PBMC Isolation and Single‐Cell RNA Sequencing

PBMCs were isolated using the Ficoll‐Paque Plus (GE Healthcare) density gradient centrifugation within 2 h of blood collection. Cell viability was confirmed (> 90%), and samples were processed using the 10x Genomics Chromium Single Cell 3′ Reagent Kit v3. Libraries were prepared according to the manufacturer’s protocol and sequenced on an Illumina NovaSeq 6000 platform. After demultiplexing and alignment to the GRCh38 reference genome using Cell Ranger (v6.1.2), count matrices were generated for downstream analysis.

### 2.3. Data Preprocessing and Integration

Initial quality control (QC), filtering, and normalization were performed using the Seurat R package [15]. Raw sequencing data were demultiplexed and aligned to the GRCh38 reference genome using Cell Ranger (v6.1.2). To ensure high‐quality data analysis, strict QC metrics were applied using the Seurat R package. Cells were retained only if they met the following criteria: (1) detection of between 200 and 6000 unique features (genes) to exclude low‐quality cells or potential doublets; (2) mitochondrial gene content < 15% to remove dying cells; and (3) a minimum of 500 unique molecular identifier (UMI) counts.

Following these filtering steps, data were normalized using the SCTransform workflow, which accounts for sequencing depth and variance. Potential batch effects were corrected during the integration of pre‐ and post‐RT datasets. Dimensionality reduction was performed via principal component analysis (PCA), and the top 30 principal components were used for uniform manifold approximation and projection (UMAP) embedding [16].

### 2.4. Cell Type Annotation

Cell types were annotated based on canonical markers and verified by reference‐based mapping using SingleR [17]. Major immune cell types were annotated based on canonical marker genes and verified using SingleR (v1.8.1) with the Human Primary Cell Atlas reference [18]. Monocyte subsets were defined using CD14 and FCGR3A expression: classical (CD14^++^CD16^−^), intermediate (CD14^++^CD16^+^), and nonclassical (CD14^+^CD16^++^) [9, 10].

### 2.5. Cell–Cell Communication Analysis

CellChat (v1.6.1) was used to infer and compare intercellular communication networks between pre‐ and postradiotherapy samples [19, 20]. Signaling strength and number of interactions were calculated for each cell type and condition. Differential signaling pathways and sender–receiver interactions were visualized via circle plots, heatmaps, and chord diagrams.

### 2.6. Gene Set Variation Analysis (GSVA)

GSVA (v1.46.0) was performed using the GSVA R package to assess pathway activity across single cells [21]. Hallmark gene sets from MSigDB were used [21–23]. Pathway scores were compared across samples and cell types using boxplots and heatmaps. Enrichment trends were interpreted in the context of radiotherapy status.

### 2.7. Metabolic Pathway Activity Analysis (scMetabolism)

Metabolic pathway analysis was performed using the scMetabolism R package (v1.0.0) [24]. KEGG‐based metabolic gene sets were used to calculate activity scores for each pathway across cells. Both raw and Z‐scaled scores were visualized via heatmaps and UMAP. Selected pathways—including glycolysis/gluconeogenesis, oxidative phosphorylation, and lysine degradation—were further compared using boxplots.

### 2.8. Developmental Plasticity Analysis (CytoTRACE)

CytoTRACE (v0.3.3) was used to estimate the developmental potential of immune cells based on transcriptional entropy and gene count [25]. Cells were scored individually, and UMAP plots were colored by CytoTRACE scores. Comparisons between pre‐ and postradiotherapy groups were visualized using boxplots.

### 2.9. Monocle3 Trajectory Inference

Pseudotime trajectories were reconstructed using Monocle3 (v1.2.9) [26]. Cells were ordered along principal graphs learned via learn_graph () and order_cells () functions. Pseudotime‐dependent genes were identified using Moran’s I spatial autocorrelation and grouped into modules via find_gene_modules (). Branch‐specific gene expression was visualized using plot_genes_branched_heatmap ().

### 2.10. Gene Ontology and Pathway Enrichment

GO and KEGG enrichment analyses were performed using the clusterProfiler package (v4.6.0) on pseudotime‐associated or regulon target genes [27]. Pathways with adjusted *p*‐values < 0.05 were considered significant.

### 2.11. Flow Cytometry Validation

Paired peripheral blood was collected pre‐ and postradiotherapy and processed within 4 h. PBMCs were isolated by Ficoll, stained at 4°C with antibodies to CD45, HLA‐DR, CD14, and CD16, and then fixed and acquired on a multicolor flow cytometer with standard compensation. Gating: singlets ⟶ live CD45^+^ ⟶ HLA‐DR^+^ monocytes (intermediate SSC); subsets defined as classical (CD14^++^CD16^-^), intermediate (CD14^++^CD16^+^), and nonclassical (CD14^+^CD16^++^).

### 2.12. ELISA Quantification of Cytokines

Paired EDTA plasma was collected pre‐ and postradiotherapy, spun (1500 g, 10 min, 4°C), aliquoted, and stored at −80°C. Cytokines (TNF‐α, IL‐6, IL‐1β) were quantified in duplicate using commercial human ELISA kits according to instruction; absorbance was read at 450/570 nm, and concentrations were calculated by 4‐parameter logistic curves.

### 2.13. Statistical Analysis

Statistical tests were performed using R (v4.2.2) and Prism (v9.5.0). For two‐group comparisons, Wilcoxon rank‐sum or paired *t*‐tests were used as appropriate. For multigroup comparisons, one‐way ANOVA with Tukey’s post hoc test was applied. *p*‐values < 0.05 were considered statistically significant.

## 3. Results

### 3.1. Radiotherapy Shifts Peripheral Immune Composition Toward Innate Inflammation

To investigate the impact of radiotherapy on systemic immunity, we performed scRNA‐seq on PBMCs collected from patients before and after thoracic radiotherapy. Unsupervised clustering and UMAP visualization identified 10 major immune cell types with comparable clustering patterns in pre‐ and post‐treatment samples (Figure 1(a)). Quantitative composition analysis revealed a pronounced shift toward innate immune dominance following radiotherapy. The proportion of neutrophils increased from 39.05% pretreatment to 47.65% post‐treatment, and monocytes also expanded substantially. In contrast, T cells declined from 20.62% to 14.13%, accompanied by a reduction in NK cells (Figure 1(b)). These compositional shifts were independently validated by flow cytometry of matched PBMCs (*n* = 6), which showed concordant increases in neutrophils and decreases in T and NK cells after radiotherapy (Figure 1(c)). Differential expression analysis further indicated functional changes in these populations. Postradiotherapy neutrophils upregulated stress and inflammatory genes such as SMAP2 [28], TAGLN2 [29], and S100P [30], while monocytes elevated transcripts including CD52 [31], IFI27 [32], and multiple mitochondrial genes (Figure 1(d)). T cells from postradiotherapy blood showed increased expression of stress markers like TP53 [33], PVT1 [34], and RPS27L [35], consistent with activation‐induced dysfunction or exhaustion. These findings demonstrate that thoracic radiotherapy remodels the peripheral immune landscape by expanding inflammatory myeloid cells (monocytes and neutrophils) and contracting the lymphocyte compartment, thereby creating an inflammatory milieu with impaired adaptive immunity. This global shift toward innate immune predominance sets the stage for downstream immune reprogramming in RIHD.

FIGURE 1Radiotherapy alters immune cell composition and transcriptional states in peripheral blood immune cells. (a) UMAP plots showing clustering of major immune cell types in PBMCs from patients before (B) and after (A) radiotherapy. Cells are annotated based on canonical marker gene expression. Numbers in parentheses indicate cell counts for each cluster. (b) Bar plots quantifying relative proportions of each major immune cell type pre‐ and postradiotherapy. Radiotherapy resulted in the expansion of neutrophils and monocytes and a concomitant reduction in T cells and NK cells. Statistical significance was assessed using a paired *t*‐test or Wilcoxon signed‐rank test depending on data distribution. ^∗^ *p*  <  0.05, ^∗∗^ *p*  <  0.01, ^∗∗∗^ *p*  <  0.001, ^∗∗∗∗^ *p*  <  0.0001. (c) Donut charts illustrating the precise percentage composition of immune cell subsets in pre‐ (top) and postradiotherapy (bottom) samples. (d) Violin plots showing representative differentially expressed genes (DEGs) in NK cells, monocytes, T cells, and neutrophils.

**Figure:**
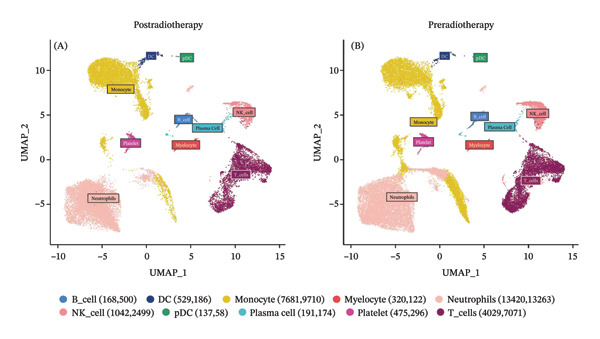
(a)

**Figure:**
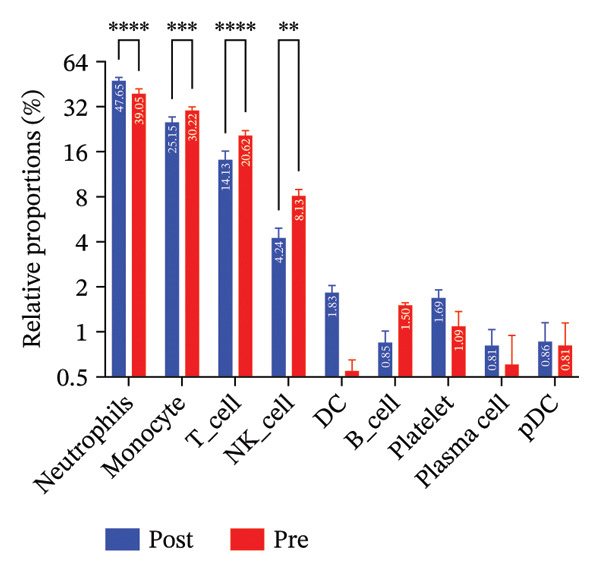
(b)

**Figure:**
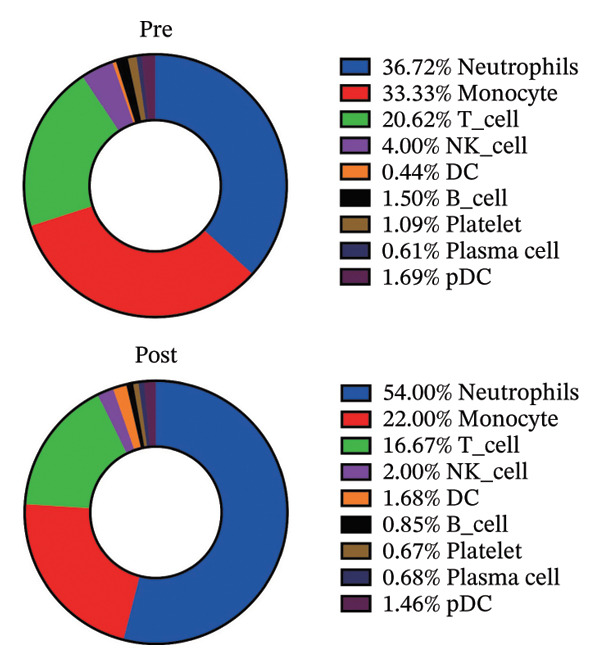
(c)

**Figure:**
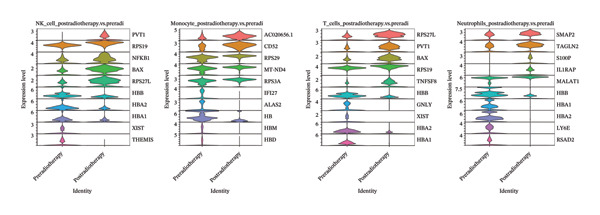
(d)

### 3.2. Radiotherapy Rewires Immune Communication Networks, Positioning Monocytes as Central Hubs

To further examine the functional consequences of radiotherapy‐induced immune remodeling, we performed intercellular communication analysis using the CellChat framework. Comparative network visualization before and after radiotherapy showed that radiation exposure substantially reconfigured cell–cell signaling interactions (Figure 2(a)). Overall, the total number of predicted ligand–receptor interactions showed a slight decrease post‐treatment (from 1267 to 1245 connections), but the aggregate interaction strength increased (overall communication strength index 29.76 pre‐ vs 30.57 postradiotherapy; Figure 2(b)). This suggests that radiotherapy amplifies immune signaling intensity and forges a more interconnected immune network despite a minor reduction in interaction quantity. At the single‐cell population level, striking shifts emerged in outgoing and incoming signaling strengths (Figure 2(c)). After radiotherapy, monocytes and neutrophils became the dominant hubs of crosstalk, exhibiting pronounced increases in both the signals they send and receive. This denotes a transition to an innate cell–centered communication network. In contrast, T cells showed marked reductions in both outgoing and incoming interaction strengths, indicating a loss of their coordinating role and suppression of adaptive immune signaling. Pairwise interaction analyses reinforced these trends: the most pronounced postradiotherapy increases in communication were between monocytes and other cell types, particularly between monocytes and T cells or NK cells (Figure 2(d)). Monocyte–T and monocyte–NK interactions not only became more frequent but also stronger, underscoring monocytes’ new role as master coordinators of the immune network. Collectively, these results indicate that radiotherapy, in addition to altering immune cell frequencies, profoundly rewires intercellular communication pathways. Monocytes emerge as central signaling nodes orchestrating the postradiotherapy immune landscape. Given this pivotal role of monocytes in the rewired network, we next examined how radiotherapy affected their internal activation states and functions.

FIGURE 2Radiotherapy enhances intercellular communication and rewires immune signaling networks. (a) Circle plots illustrating predicted ligand–receptor interactions among major immune cell types before and after radiotherapy. Node size reflects the number of interactions; edge thickness indicates interaction strength. (b) Bar plots comparing the total number (A) and aggregate strength (B) of intercellular interactions pre‐ and postradiotherapy. Post‐treatment samples show a modest reduction in the total interaction number but a significant increase in the overall interaction strength (*p*  <  0.05). (c) Scatter plots of the outgoing (*x*‐axis) versus incoming (*y*‐axis) interaction strength for each cell type. Circle size indicates the total interaction number. Monocytes and neutrophils emerged as dominant broadcasters and receivers of immune signals after radiotherapy. (d) Bar plots ranking signaling pathways based on change in the relative information flow. Proinflammatory pathways including MIF, CXCL, IL6, and CCL were upregulated, while adaptive immunity–associated pathways such as CD40, CD48, and LCK were suppressed post‐treatment. (e) Chord diagrams depicting global ligand–receptor–mediated communication networks before (A) and after (B) radiotherapy. Segments represent cell types; connecting lines indicate predicted interactions. Monocytes, neutrophils, and dendritic cells became key communication hubs post‐treatment.

**Figure:**
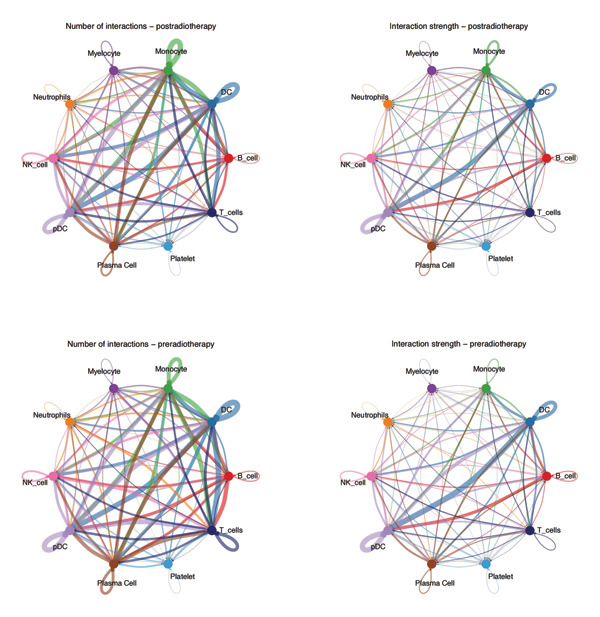
(a)

**Figure:**
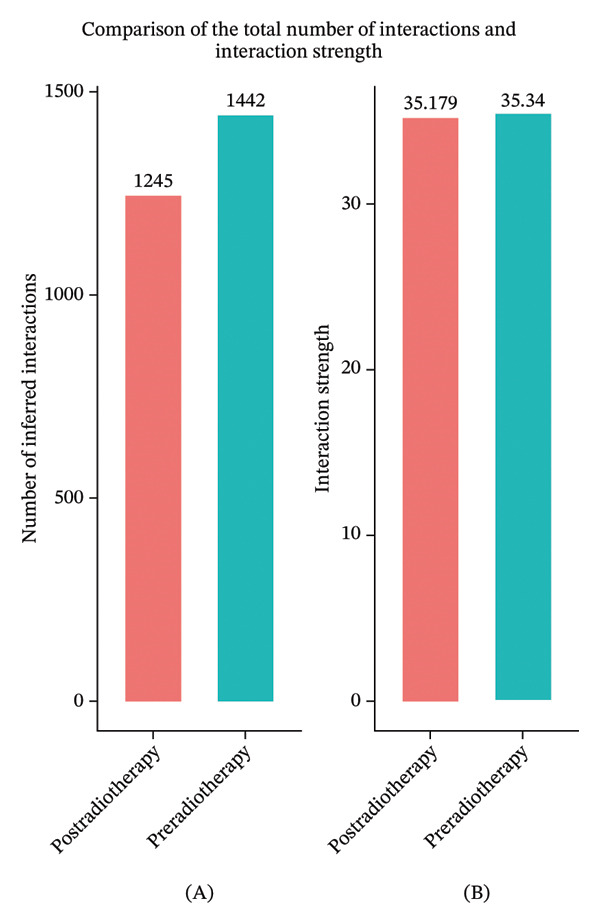
(b)

**Figure:**
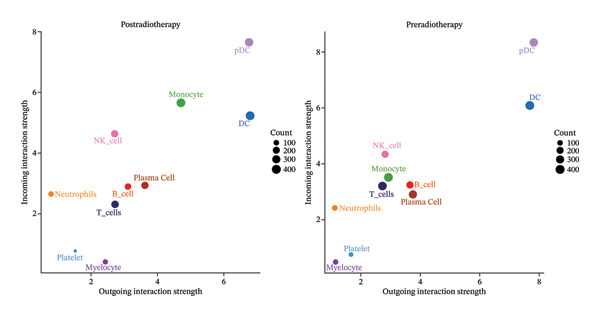
(c)

**Figure:**
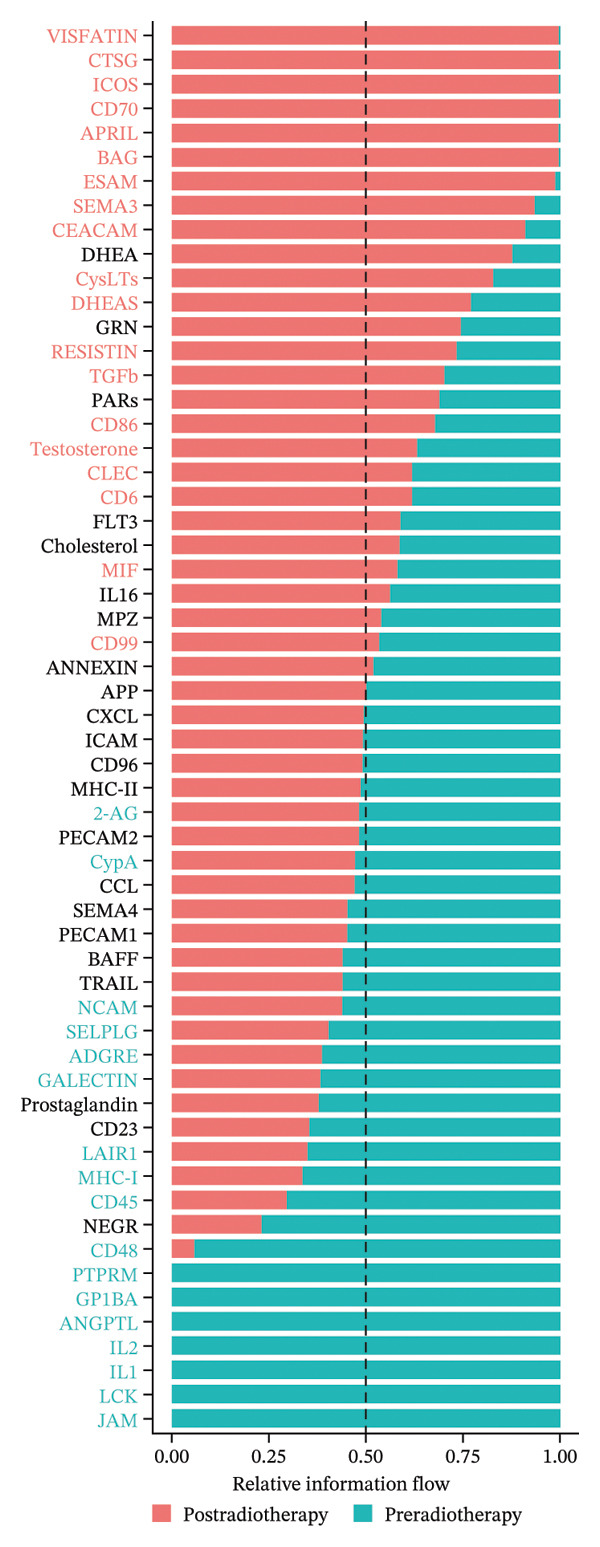
(d)

**Figure:**
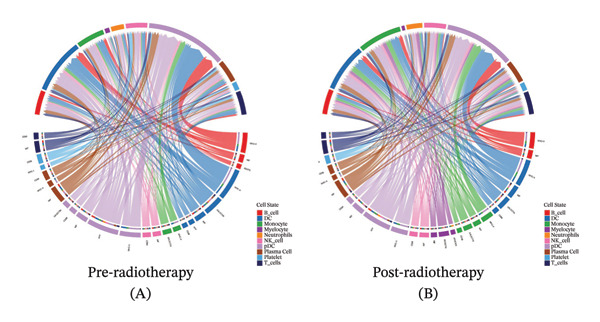
(e)

### 3.3. Radiotherapy Induces Inflammatory Signaling Pathways in Monocytes

We next investigated whether the immune compositional and communication changes were accompanied by intrinsic functional reprogramming of monocytes. GSVA of hallmark pathways in each immune subset revealed that monocytes were the most responsive cell type to radiotherapy. A heatmap of pathway enrichment scores across all major cell types demonstrated global upregulation of inflammatory, stress‐response, and metabolic pathways in postradiotherapy samples, with monocytes showing the highest overall increase in pathway activity (Figure 3(a)). Focusing on monocytes, we identified the top pathways upregulated after radiotherapy (Figure 3(b)). This list included canonical proinflammatory signaling cascades such as IL‐6–JAK–STAT3 [36], TNF–NF‐κB [37], and TGF‐β [38] signaling, alongside pathways reflecting cellular growth and stress adaptation (e.g., mTORC1 signaling [39], MYC targets [40], and the unfolded protein response (UPR) [41]). The enrichment of these pathways suggests that radiotherapy triggers a multifaceted activation program in monocytes.

FIGURE 3Radiotherapy induces inflammatory and metabolic reprogramming in monocytes. (a) Heatmap of GSVA pathway enrichment scores across major immune cell types, showing postradiotherapy upregulation of inflammatory and metabolic signaling pathways in monocytes. (b) Top 10 hallmark pathways most upregulated in monocytes after radiotherapy, ranked by the mean GSVA score increase. (c) Violin plots of enriched inflammatory signaling pathways in monocytes: IL6–JAK–STAT3, TGF‐β signaling, and TNF–NFκB signaling. (d) Violin plots showing inflammatory cytokine expression of IL‐6, IL1‐β, TNF‐α in patient by ELISA. (e) Violin plots showing metabolic pathway enrichment: mTORC1 signaling, MYC targets, and unfolded protein response (UPR) reflecting biosynthetic and stress‐adaptive program activation. (f) Boxplots showing single‐cell inferred metabolic activity from scMetabolism analysis. Glycolysis/gluconeogenesis and glutathione metabolism were significantly increased in monocytes postradiotherapy, while oxidative phosphorylation was modestly reduced. Statistical significance was calculated using the Wilcoxon rank‐sum test. ^∗^ *p*  <  0.05, ^∗∗^ *p*  <  0.01, ^∗∗∗^ *p*  <  0.001, ^∗∗∗∗^ *p*  <  0.0001.

**Figure:**
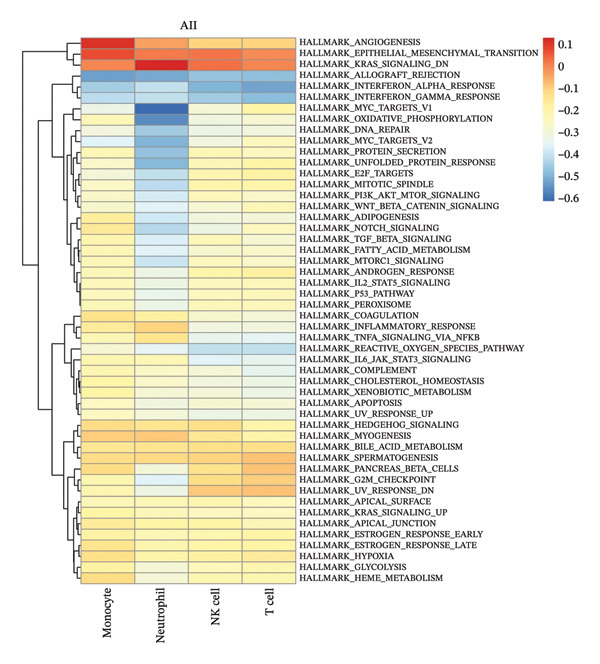
(a)

**Figure:**
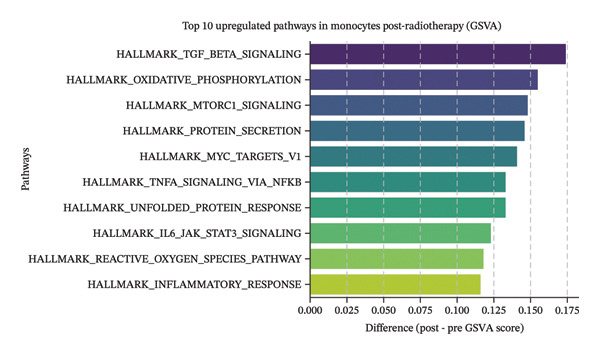
(b)

**Figure:**
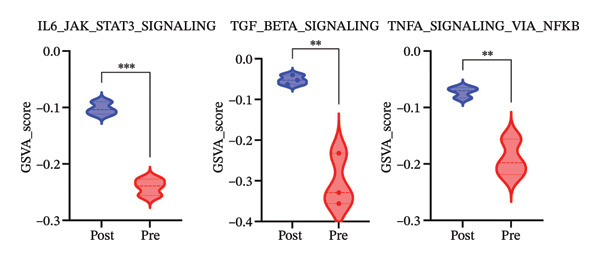
(c)

**Figure:**
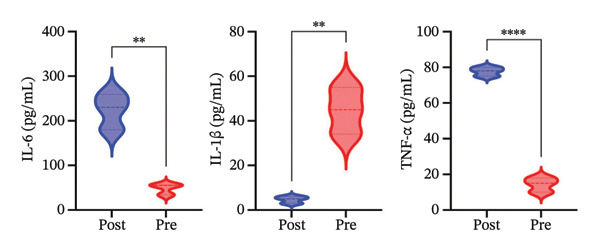
(d)

**Figure:**
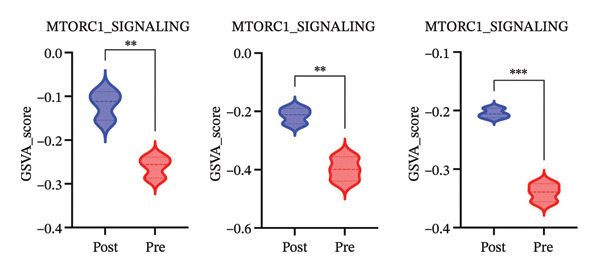
(e)

**Figure:**
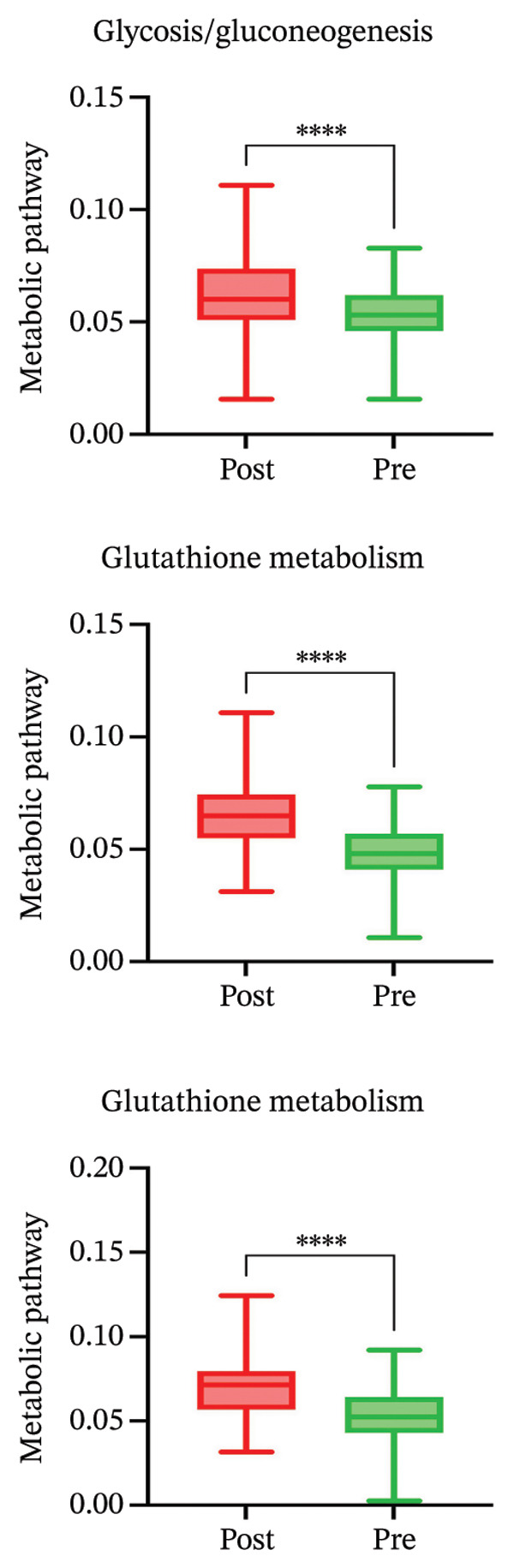
(f)

We further validated key components of this program at the single‐cell level. Violin plots confirmed robust post‐treatment activation of IL‐6–JAK–STAT3 signaling [36], TGF‐β signaling [38], and TNF‐α–NF‐κB signaling [37] (Figure 3(c)) in monocytes. These pathways are well‐known orchestrators of monocyte activation and inflammatory cytokine production, driving recruitment of additional leukocytes and polarization of the immune response toward a proinflammatory state. In summary, radiotherapy exposure launches a broad proinflammatory transcriptional program in circulating monocytes, characterized by the induction of cytokine signaling and stress‐response pathways that can potentiate systemic inflammation. Notably, several of the upregulated pathways (such as mTORC1 and MYC‐driven transcription) are closely tied to cellular metabolic activity, hinting that monocytes may undergo metabolic reconfiguration in parallel with these inflammatory changes.

To provide orthogonal protein‐level validation of this inflammatory program, we quantified circulating cytokines in matched pre‐ and postradiotherapy samples by ELISA (Figure 3(d)). Consistent with the transcriptomic activation of cytokine‐related pathways in monocytes, IL‐6 and TNF‐α protein concentrations were significantly increased after radiotherapy, whereas IL‐1β levels were significantly reduced. Together, these data support that thoracic radiotherapy is accompanied by a measurable shift in the systemic inflammatory milieu while also highlighting that individual cytokines can exhibit distinct regulation/kinetics at the protein level relative to transcript‐level pathway activation.

### 3.4. Radiotherapy Drives Metabolic Reprogramming in Monocytes

In parallel with the heightened inflammatory signaling, monocytes exhibited clear signs of metabolic reprogramming after radiotherapy. Several pathways related to cellular metabolism and biosynthesis were among the top upregulated programs in postradiotherapy monocytes (Figure 3(b) in the prior section). In particular, strong enrichment of mTORC1 signaling [39] (a central regulator of nutrient sensing and anabolic metabolism), MYC targets [40] (which drive glycolytic and proliferative programs), and the UPR [41] (which is activated by endoplasmic reticulum stress) was observed in monocytes (Figure 3(e)). The concurrent activation of these metabolic regulators suggests that radiotherapy not only triggers inflammatory gene expression but also shifts monocytes toward a state of high metabolic activity to support their functional changes. This raised the possibility that monocytes adapt their metabolic wiring (e.g., energy production and redox balance) to meet the demands of a proinflammatory, proliferative phenotype under radiotherapy stress.

To directly evaluate functional metabolic changes, we applied single‐cell metabolic flux analysis using the scMetabolism toolkit. Globally, monocytes showed the most pronounced metabolic alterations among all immune subsets following radiotherapy (Supporting Figure S1A). Finer analysis of monocyte‐specific metabolism (Supporting Figure S1B) revealed selective upregulation of pathways involved in energy production and oxidative stress mitigation, including glycolysis/gluconeogenesis, glutathione metabolism, and fatty acid degradation. These changes highlight an increase in metabolic plasticity, enabling monocytes to generate ATP through glycolytic pathways and to enhance their antioxidant capacity.

Quantitative comparisons confirmed that postradiotherapy monocytes had significantly elevated glycolytic activity (Figure 3(f)), indicative of a shift toward aerobic glycolysis (Warburg‐like metabolism) to rapidly fuel immune effector functions [42]. At the same time, glutathione metabolism was markedly increased, reflecting an upregulated antioxidant defense likely aimed at counteracting radiation‐induced oxidative stress [43]. Interestingly, oxidative phosphorylation showed a mild but consistent reduction in activity in post‐treatment monocytes, which could indicate mitochondrial remodeling or a diversion of resources toward glycolysis [44].

Together, these data delineate a coordinated transcriptional and metabolic remodeling of monocytes in response to radiotherapy. Monocytes not only activate inflammatory pathways but also rewire their metabolism—boosting glycolytic flux and antioxidant defenses—to sustain and survive their heightened functional state. This comprehensive reprogramming reinforces the central role of monocytes in shaping the postradiotherapy immune environment and suggests that they are actively adapting to the metabolic and oxidative challenges imposed by radiation.

### 3.5. Monocytes Exhibit the Highest Transcriptional Plasticity and Serve as the Primary Responders to Radiotherapy‐Induced Reprogramming

Given the broad activation of monocytes observed, we next asked whether radiotherapy also alters the developmental or differentiation state of immune cells. We applied CytoTRACE analysis to quantify the developmental potential of each immune cell, as CytoTRACE scores inversely correlate with differentiation (higher score = more progenitor‐like). This revealed substantial heterogeneity in differentiation states across cell types (Figure 4(a)). Monocytes and T cells exhibited elevated CytoTRACE scores, reflecting a less differentiated and more transcriptionally plastic state, in contrast to neutrophils, which predominantly displayed low scores indicative of terminal maturation. Notably, monocytes demonstrated the broadest score distribution across the full differentiation continuum, suggesting the presence of diverse intermediate states. This continuous developmental spectrum places monocytes among the most plastic immune populations (Figure 4(b)), highlighting their dynamic capacity for state transitions in response to radiotherapy‐induced stress.

FIGURE 4Radiotherapy promotes developmental plasticity remodeling in monocytes. (a) Uniform Manifold Approximation and Projection (UMAP) plot colored by CytoTRACE score (A) and by annotated immune cell types (B), including T cells, NK cells, monocytes, and neutrophils. CytoTRACE scores reflect the predicted differentiation state of individual cells, with higher values (red) indicating lower differentiation potential and greater transcriptional plasticity. (b) Boxplot comparing CytoTRACE scores among the four major immune populations. T cells and monocytes exhibited relatively higher CytoTRACE scores. Statistical significance was calculated using the Wilcoxon rank‐sum test, with neutrophils used as the reference group. (c) UMAP visualization of CytoTRACE scores (A) and treatment groups (B), comparing pre‐ and postradiotherapy of monocytes. Cells from postradiotherapy samples were enriched in regions with higher CytoTRACE scores, especially within the monocyte compartment, implying a radiation‐induced shift toward a more plastic or progenitor‐like transcriptional profile. (d) Boxplot comparing CytoTRACE scores between preradiotherapy and postradiotherapy of monocytes. Postradiotherapy cells displayed significantly higher CytoTRACE scores, and intermediate monocytes postradiotherapy were compared to all monocytes preradiotherapy. (e) UMAP plot showing the classification of monocyte subtypes into classical, intermediate, and nonclassical monocytes. Cell numbers for each subset are indicated. (f) Bar plot displaying the proportion of preradiotherapy and postradiotherapy cells within each monocyte subset. Postradiotherapy samples show a striking enrichment of intermediate monocytes from 6.33% to 18.33%. (g) Bar plot displaying the proportion of preradiotherapy and postradiotherapy cells within each monocyte subset by flow cytometry. Postradiotherapy samples show a striking enrichment of intermediate monocytes from 9.67% to 23.67%. (h) CytoTRACE projection (A) and monocyte subtype annotation (B) on the same UMAP space. Intermediate monocytes were concentrated in high CytoTRACE score regions, indicating lower differentiation and greater transcriptional plasticity. (i) Boxplot comparing CytoTRACE scores among the three monocyte subtypes. Intermediate monocytes exhibit the highest CytoTRACE scores, suggesting they are the least differentiated and most plastic among circulating monocytes. Nonclassical monocytes served as the reference group for pairwise comparisons with intermediate and classical subsets. All statistical significance was calculated using the Wilcoxon rank‐sum test. ^∗^ *p*  <  0.05, ^∗∗^ *p*  <  0.01, ^∗∗∗^ *p*  <  0.001, ^∗∗∗∗^ *p*  <  0.0001.

**Figure:**
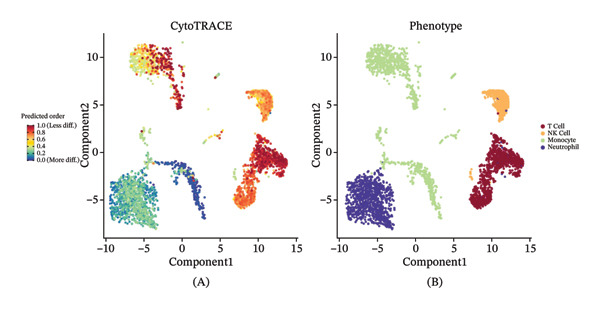
(a)

**Figure:**
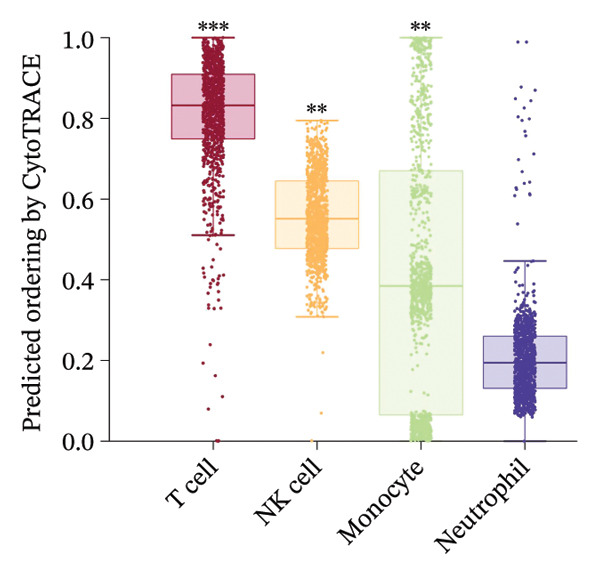
(b)

**Figure:**
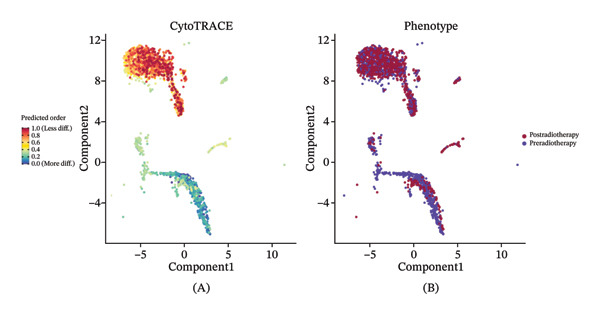
(c)

**Figure:**
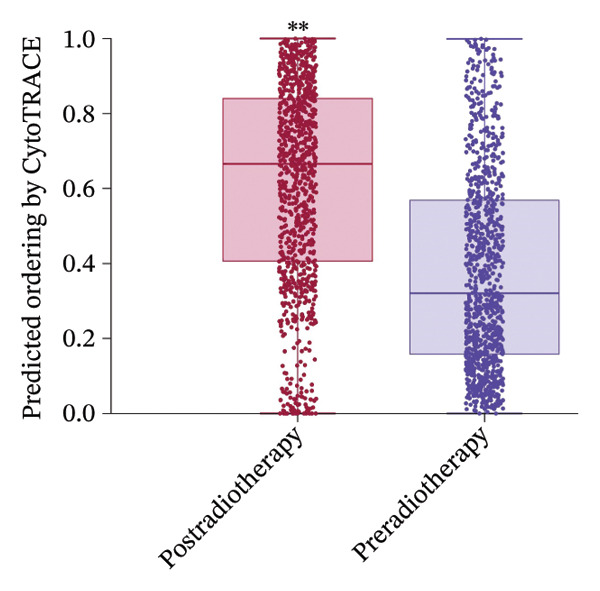
(d)

**Figure:**
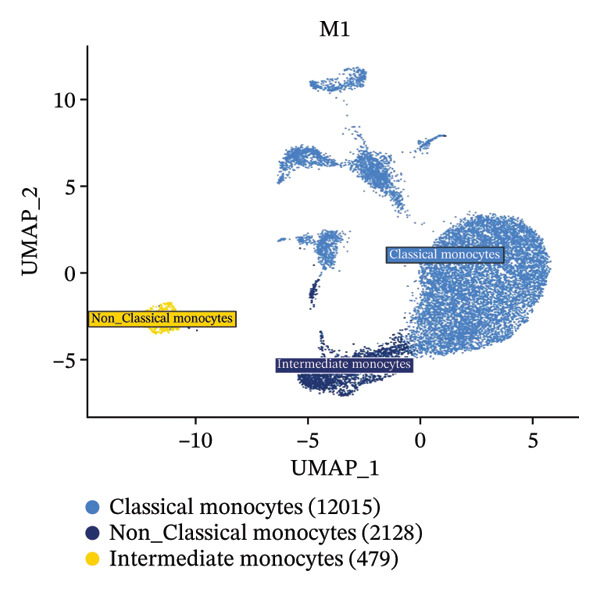
(e)

**Figure:**
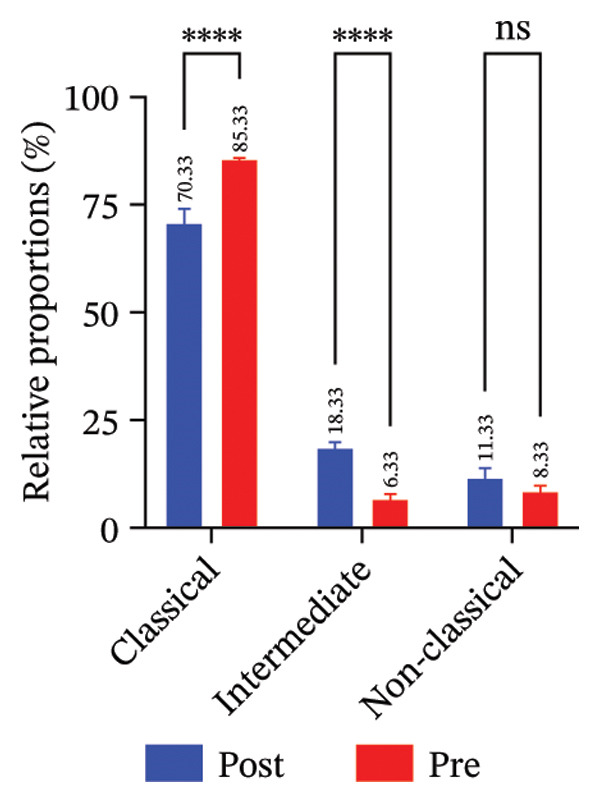
(f)

**Figure:**
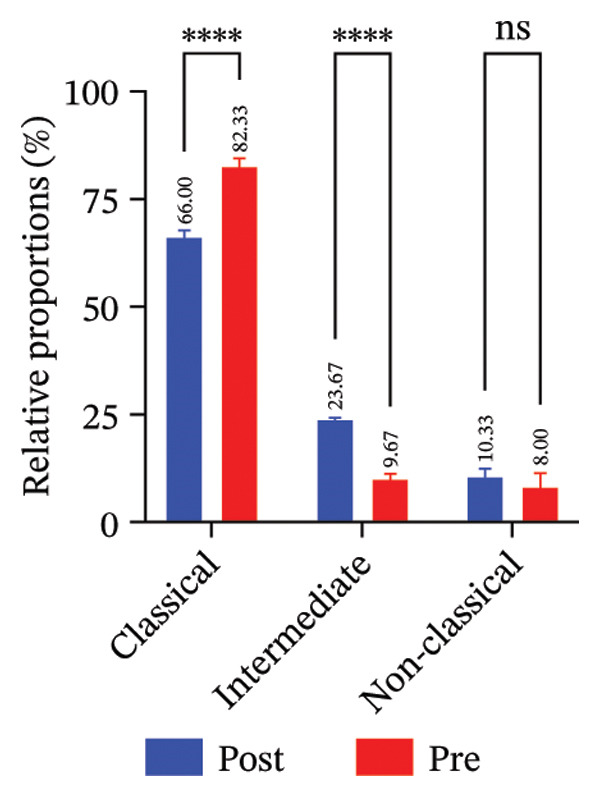
(g)

**Figure:**
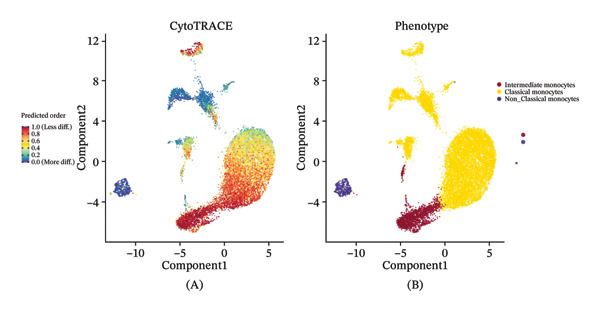
(h)

**Figure:**
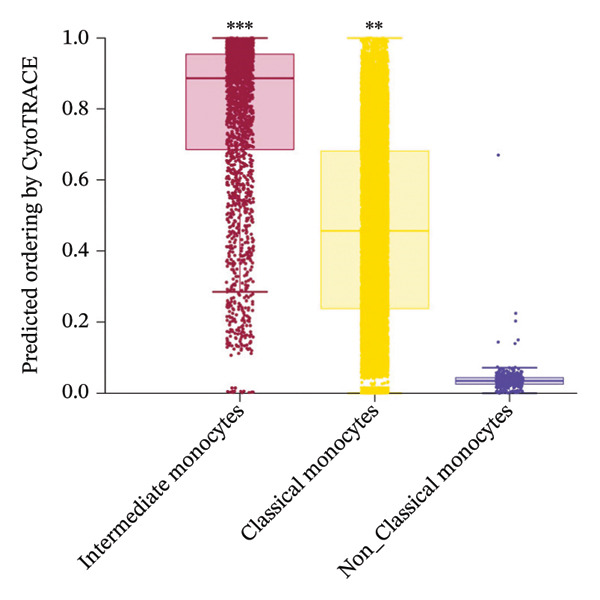
(i)

We then compared the overall CytoTRACE score distributions in pre‐ versus postradiotherapy samples to determine if radiotherapy skews the developmental landscape of circulating immune cells. Both pre‐ and post‐treatment cells spanned a range of differentiation states (Supporting Figure S2A); however, cells from postradiotherapy samples were visibly enriched in regions of the UMAP corresponding to high CytoTRACE values. Accordingly, the average CytoTRACE score across all immune cells was significantly elevated after radiotherapy (Supporting Figure S2B), indicating a systemic shift toward less differentiated states in the immune compartment. In other words, radiation exposure preserved or promoted more progenitor‐like transcriptional programs in circulating immune cells. This effect was most pronounced in monocytes.

Focusing on the monocyte subset, we observed that monocytes from postradiotherapy blood clustered in UMAP regions associated with higher CytoTRACE scores, often overlapping early pseudotime positions (Figure 4(c)). Quantitatively, postradiotherapy monocytes had significantly greater CytoTRACE scores than their pretreatment counterparts (Figure 4(d)). This radiation‐associated increase in monocyte developmental potential suggests that monocytes were maintained in a more undifferentiated, plastic state following exposure. Taken together, these findings indicate that monocytes are not only inherently more transcriptionally plastic than many other immune cells, but that radiotherapy further enhances this plasticity. Monocytes thus emerge as primary responders that retain stem‐like properties under radiotherapy, positioning them as key intermediaries for downstream immune reconfiguration.

### 3.6. Radiotherapy Preferentially Expands the Highly Plastic Intermediate Monocyte Subset

To further resolve the monocyte compartment’s heterogeneity, we stratified circulating monocytes into their three established subsets: classical (CD14^++^CD16^−^), intermediate (CD14^++^CD16^+^), and nonclassical (CD14^+^CD16^++^). Strikingly, the intermediate subset increased from 6.33% of total monocytes pretreatment to 18.33% post‐treatment, accompanied by a reciprocal decrease in classical monocytes, while nonclassical cells were largely unchanged (Figures 4(e) and 4(f)). Multiparameter flow cytometry confirmed the same pattern, with intermediate monocytes rising and classical monocytes falling and nonclassical remaining nonsignificant (Figure 4(g)). This shift indicates that radiotherapy preferentially expands a transcriptionally dynamic subpopulation known for its responsiveness.

Intermediate monocytes are characterized by high expression of activation markers and are often considered a transitional state; their expansion suggests an accumulation of cells in a less differentiated, highly reactive phase of the monocyte lineage. Indeed, CytoTRACE analysis within the monocyte pool revealed that intermediate monocytes localized to regions of the UMAP associated with low differentiation (high CytoTRACE) scores (Figure 4(h)). When comparing CytoTRACE metrics between monocyte subsets, intermediate monocytes displayed the highest developmental plasticity of all (Figure 4(i)), higher than both classical and nonclassical subsets. This confirms that the intermediate subset inherently represents a more progenitor‐like and plastic state.

Radiotherapy not only skewed subset frequencies toward these intermediate monocytes but also appeared to maintain monocytes in a less mature state. Comparing monocyte CytoTRACE scores between treatment conditions, we found that postradiotherapy monocytes overall had higher scores than preradiotherapy monocytes (Supporting Figures S2C and S2D), consistent with a radiation‐driven delay in monocyte terminal differentiation. In summary, thoracic radiotherapy imposes a robust and selective remodeling of the circulating monocyte compartment. The intermediate monocyte subset, distinguished by its high plasticity, is preferentially expanded and retains an undifferentiated state after radiation. These intermediate monocytes likely form the transcriptionally plastic core that mediates downstream inflammatory and functional adaptations to radiotherapy.

### 3.7. Radiotherapy Accelerates Intermediate Monocyte Differentiation Into Inflammatory Phenotypes

Building on the observation that intermediate monocytes expand and harbor high developmental potential postradiotherapy, we next investigated their fate using pseudotime trajectory analysis. We constructed a differentiation trajectory for monocytes with Monocle3 to determine how intermediate and classical subsets relate in a developmental timeline. The inferred trajectory revealed a continuous path initiating with intermediate monocytes at the early pseudotime stages and progressing toward terminally differentiated classical monocytes at the late stages (Figure 5(a)). This ordering is consistent with the model that intermediate monocytes are precursors that mature into classical monocytes. Notably, radiotherapy was associated with a clear perturbation of this trajectory. In postradiotherapy samples, the relative abundance of intermediate monocytes along the early pseudotime was markedly reduced, whereas classical monocytes were enriched at the terminal pseudotime end (Figure 5(b)). In other words, after radiotherapy, cells were redistributed further along the differentiation axis, suggesting that the transition from intermediate to classical monocyte state was accelerated. This shift implies that radiation exposure drives intermediate monocytes to more rapidly differentiate into an activated, end‐stage monocyte phenotype, which we hypothesize to be proinflammatory.

FIGURE 5Pseudotime trajectory and functional rewiring of monocyte differentiation after radiotherapy. (a) UMAP visualization displaying the pseudotime developmental trajectory of monocyte subsets. Cells are colored according to their subtype identity: classical monocytes (red) and intermediate monocytes (blue). Black lines represent the inferred trajectory paths derived from Monocle3 pseudotime analysis. (b) Comparison of monocyte subset distributions along the pseudotime trajectory before (preradiotherapy, right panel) and after radiotherapy (postradiotherapy, left panel). (c) Dynamic gene expression patterns of monocyte subset markers along pseudotime trajectories. Scatter plots illustrate the expression levels of representative monocyte markers across pseudotime (*x*‐axis), reflecting differentiation from intermediate (blue dots) to classical monocytes (red dots). Fitted curves (black lines) summarize expression trends across pseudotime. (d) Gene Ontology (GO) bar plot showing top enriched biological processes of genes within Module 1, primarily associated with immune activation, cell adhesion, and monocyte differentiation. The color gradient represents the significance level (−log10 *p.adjust*), and bar length indicates gene count. (e) KEGG pathway enrichment bar plot of Module 1 genes highlighting key immune‐related signaling pathways such as cytokine–cytokine receptor interaction, PI3K–Akt, and MAPK signaling. (f) GO enrichment analysis of genes in Module 2. Top enriched biological processes include reactive oxygen species (ROS) metabolic process, hydrogen peroxide catabolism, antioxidant activity, and hemoglobin/oxygen carrier complex involvement. (g) KEGG pathway enrichment of Module 2 genes. Although limited in pathway count, significant enrichment was observed in malaria and African trypanosomiasis pathways.

**Figure:**
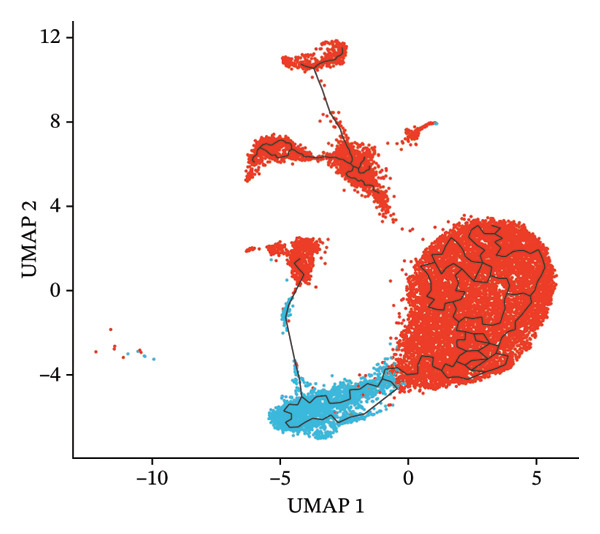
(a)

**Figure:**
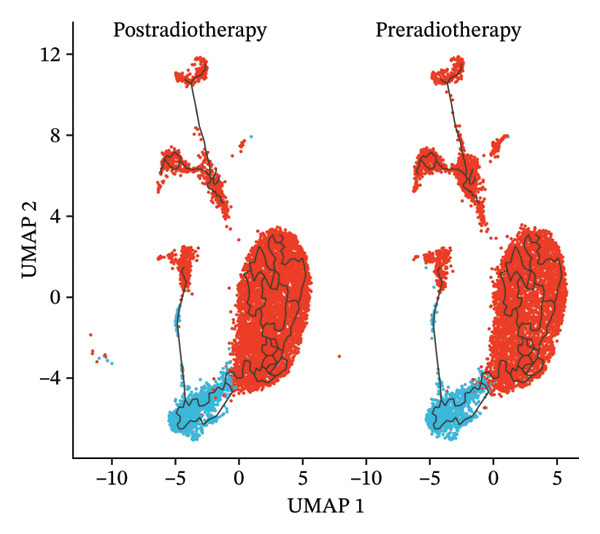
(b)

**Figure:**
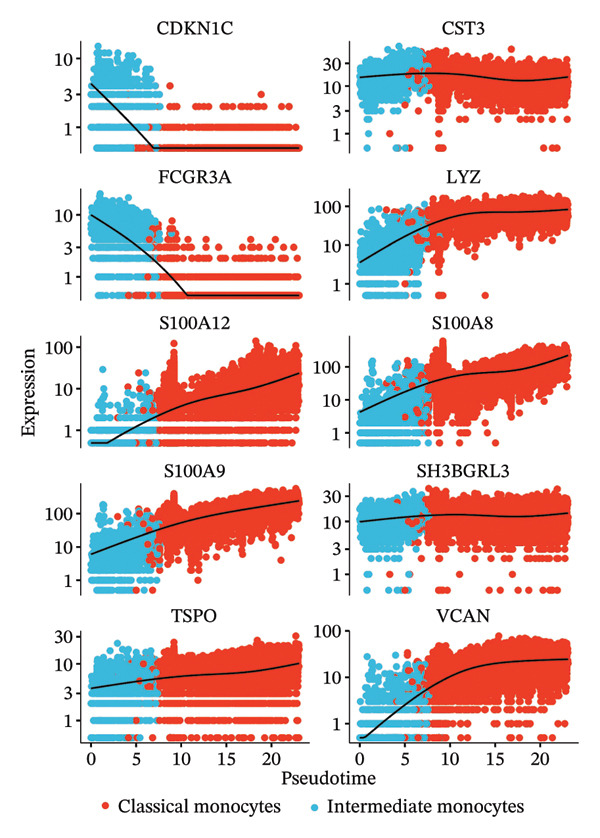
(c)

**Figure:**
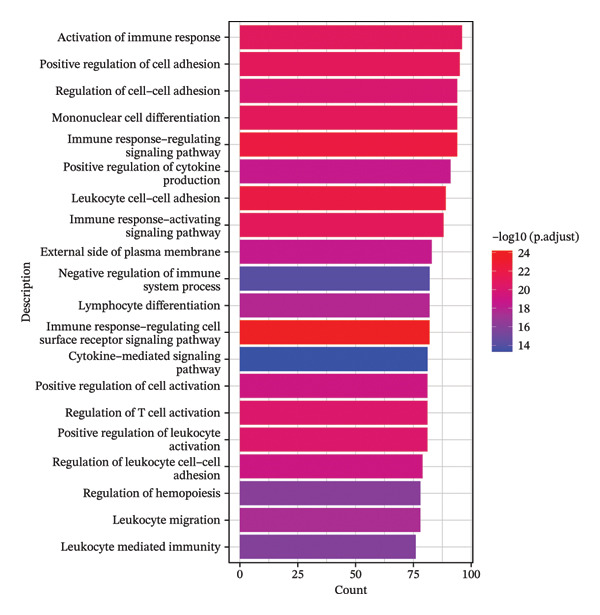
(d)

**Figure:**
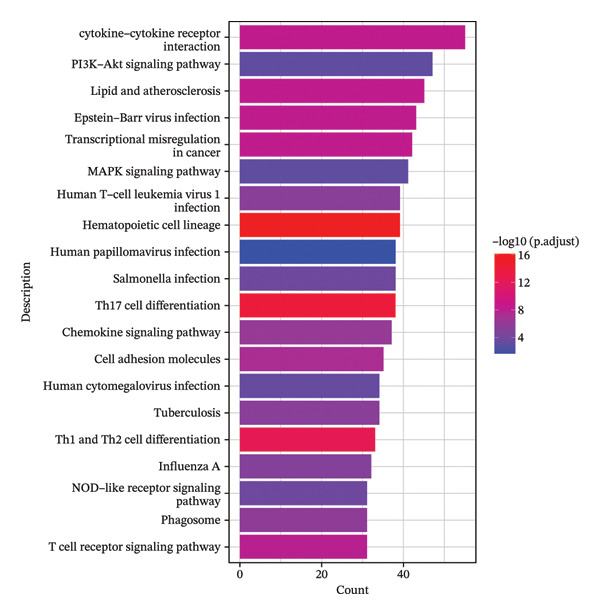
(e)

**Figure:**
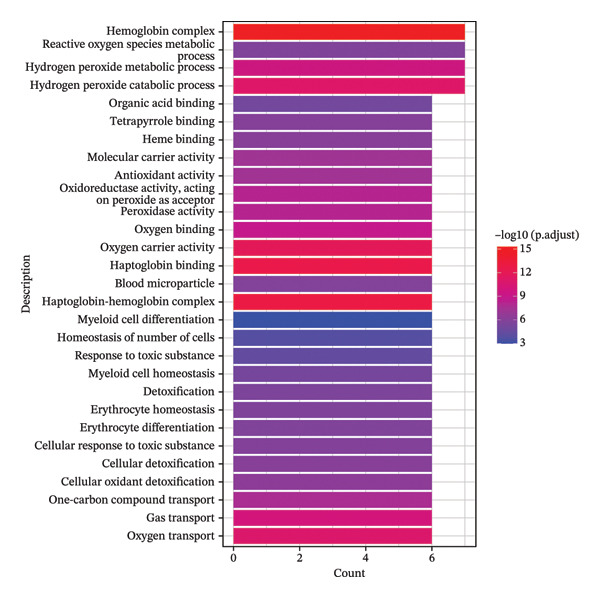
(f)

**Figure:**
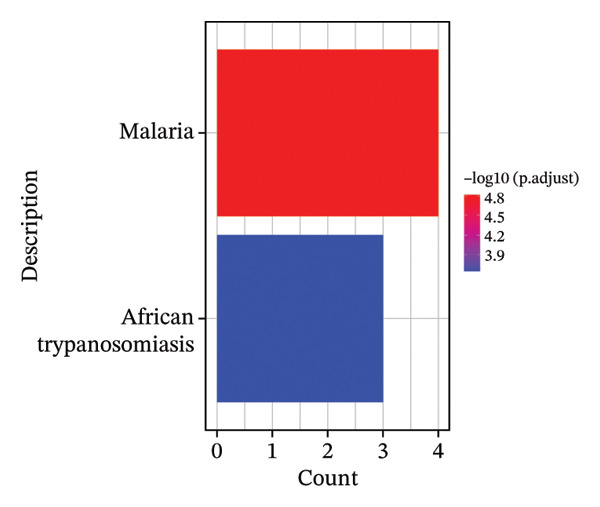
(g)

To validate this, we examined gene expression dynamics along the pseudotime trajectory. We found that key genes characteristic of the intermediate state were highly expressed at the beginning of the trajectory and then downregulated as cells progressed. For instance, FCGR3A [45] (encoding CD16) and CDKN1C [46] (p57/KIP2, a cell cycle regulator)—markers associated with intermediate monocytes—showed a gradual decline along pseudotime. By contrast, genes associated with classical monocytes and inflammatory activation became progressively upregulated toward the end of the trajectory. Notably, S100A8 [11], S100A9 [47], and S100A12 [48] (which encode calprotectin subunits and related alarmin proteins) were sharply elevated in the late‐pseudotime cells, as were other classical monocyte genes such as LYZ (lysozyme), VCAN (versican), TSPO, and SH3BGRL3 (Figure 5(c)). The increasing expression of S100A8/A9/A12 and other inflammatory mediators confirms that as intermediate monocytes convert into classical monocytes, they acquire a pronounced proinflammatory gene signature. The pseudotime gene expression patterns, coupled with the altered distribution of cells along the trajectory, highlight that intermediate monocytes serve as transitional precursors that give rise to inflammatory classical monocytes following radiotherapy. In essence, radiation pushes the intermediate monocytes to differentiate “ahead of schedule” into an activated state enriched in proinflammatory effectors, which provides a mechanistic link between the expansion of intermediate monocytes and the escalation of systemic inflammation in RIHD.

### 3.8. Radiotherapy Engages Bifurcated Monocyte Programs for Inflammation and Oxidative Stress Adaptation

While the trajectory analysis indicated that inflammatory differentiation is a dominant fate for monocytes postradiotherapy, we next explored whether radiotherapy also invokes additional adaptive programs in monocytes, beyond inflammatory activation. We performed pseudotime‐based gene module clustering to unbiasedly identify coregulated gene sets along the monocyte developmental trajectory. This analysis uncovered two distinct gene modules that define bifurcated transcriptional programs during monocyte maturation under radiotherapy exposure.

Module 1 consisted of genes whose expression increased steadily toward the classical monocyte endpoint of the trajectory. Functional enrichment analysis of these genes revealed a strong immunological theme. Gene Ontology terms such as “cytokine‐mediated signaling pathway,” “mononuclear cell differentiation,” and “leukocyte adhesion” were significantly enriched (Figure 5(d)), indicating that Module 1 genes are involved in immune activation and effector functions. Correspondingly, KEGG pathway analysis showed that Module 1 was enriched in pathways like cytokine–cytokine receptor interactions, PI3K–Akt signaling [49], and Th17 cell differentiation [50] (Figure 5(e)), all of which are associated with promoting inflammation and adaptive immune responses. These findings reinforce that one arm of the radiotherapy‐altered monocyte program is geared toward driving robust inflammatory and immunostimulatory activity as monocytes mature.

In contrast, Module 2 comprised genes that peaked in expression at early‐to‐mid pseudotime and then declined as cells reached the terminal classical state. This module’s gene functions were distinct, centering on cellular stress responses. Enriched Gene Ontology terms for Module 2 included “response to oxidative stress,” “hydrogen peroxide metabolic process,” “heme binding,” and “oxidoreductase activity” (Figure 5(f)). KEGG analysis further highlighted pathways related to redox homeostasis and antioxidant defense, such as glutathione metabolism and peroxisome pathways (Figure 5(g)). The temporal pattern of Module 2 gene expression—high in less differentiated monocytes and lower in fully differentiated ones—suggests that monocytes transiently activate a stress‐mitigation program as they begin to mature under postradiotherapy conditions. This likely represents a protective, stress‐adaptive response to the elevated oxidative burden and DNA damage imposed by radiation. Monocytes appear to invoke antioxidant and detoxification mechanisms in parallel with their inflammatory programming, perhaps to enable survival and function in a harsh postradiation environment.

In summary, our analysis supports a dual‐program model of monocyte reprogramming after radiotherapy: one program drives inflammatory activation and immune effector differentiation, while the other confers resistance to oxidative stress and maintains cellular homeostasis. These bifurcated transcriptional programs operate concurrently, reflecting the remarkable plasticity of intermediate monocytes in balancing multiple functional demands. Intermediate monocytes thus act not only as precursors of proinflammatory cells but also as metabolic sensors and stress regulators capable of mounting compensatory antioxidant responses. This parallel engagement of inflammatory and stress‐adaptive pathways underscores the complex, adaptive nature of the monocyte response in the context of RIHD. Radiotherapy essentially reprograms intermediate monocytes to walk two paths at once: fueling systemic inflammation, on one hand, while mitigating radiation‐induced damage, on the other.

## 4. Discussion

Thoracic radiotherapy has long been known to provoke inflammatory reactions, but our single‐cell analysis provides a detailed peripheral, high‐resolution view of how the immune system—and monocytes in particular—is remodeled after thoracic radiotherapy. We did not analyze cardiac tissue; therefore, inferences are limited to peripheral immune remodeling rather than myocardial mechanisms. A central finding of this study is that intermediate monocytes appear to become central correlates in the postradiotherapy immune landscape. We observed an expansion of this CD14^++^CD16^+^ subset alongside broad transcriptomic changes that are consistent with these cells acting as inflammation response and active responders to oxidative stress. Collectively, our results suggest a peripheral, RT‐associated innate immune response wherein intermediate monocytes may serve as a potential circulating correlate linking radiation exposure to systemic inflammation. We hypothesize that these peripheral changes could reflect or contribute to the subsequent cardiac injury in RIHD.

Our data in PBMC demonstrate that radiotherapy exposure skews the peripheral immune system toward an innate‐dominant, proinflammatory state. This is evidenced by the increased frequency of monocytes and neutrophils and the concomitant decline in lymphoid cells after treatment. These shifts align with clinical observations that radiation can cause lymphopenia and innate immune activation. Indeed, recent studies have shown that ionizing radiation directly activates monocytes and macrophages through cell‐intrinsic DNA/RNA damage sensing pathways, leading to the production of type I interferons and inflammatory cytokines [51]. Our finding of enhanced IL‐6–STAT3 and NF‐κB signaling in postradiotherapy monocytes is consistent with radiation triggering such innate immune sensors and downstream inflammatory programs. Moreover, we found monocytes to be central hubs in cell–cell communication networks after radiotherapy, strongly engaging in crosstalk with other immune cells (particularly T and NK cells). This indicates that monocytes not only are activated themselves but also actively coordinate the immune response by emitting and receiving signals that can amplify inflammation and modulate lymphocyte function. Monocyte‐derived signals (e.g. IL‐6, TNF‐α, CXCL chemokines) can promote recruitment of additional myeloid cells and inhibit effective T‐cell responses, which may explain the immunosuppressive yet inflammatory milieu (often termed “inflamed immune suppression”) that we observed following radiotherapy. Notably, radiation‐induced damage‐associated molecular patterns such as extracellular CIRP have been shown to amplify monocyte/macrophage activation via receptors like TREM‐1, providing one potential mechanism for the heightened intercellular signaling we report [52]. Thus, our results place monocytes at the center of a radiation‐provoked inflammatory network, in agreement with the emerging literature on radiation’s capacity to rewire innate immunity.

A novel insight from our study is the identification of intermediate monocytes as critical mediators of the radiotherapy‐induced immune reprogramming. Intermediate monocytes were not only overrepresented after treatment, but they also exhibited the highest developmental plasticity and were enriched at the origin of monocyte differentiation trajectories. In baseline conditions (e.g., in healthy or pr‐treatment blood), intermediate monocytes are known to possess a proinflammatory profile and serve as an intermediary stage that can give rise to other monocyte subsets or macrophages [10]. Their propensity to secrete inflammatory cytokines and present antigens positions them as potent effectors in immune responses. Our findings suggest that radiation augments these inherent properties—essentially “priming” intermediate monocytes to rapidly differentiate and release a surge of inflammatory mediators. The pseudotime analysis revealed that postradiotherapy intermediate monocytes quickly transition into classical monocytes that are transcriptionally enriched for alarmins like S100A8/A9/A12, as well as other inflammatory molecules. S100A8/A9 (calprotectin) in particular is a proinflammatory damage‐associated molecular pattern that can drive cytokine release and leukocyte recruitment. The upregulation of S100A8/A9 we observed in monocytes after radiotherapy is therefore a strong indicator of a feed‐forward inflammatory loop being established [47]. This observation is concordant with the role of S100A8/A9 as a key inflammatory mediator and biomarker across cardiovascular conditions. This is significant because elevated intermediate monocytes and S100A8/A9 have been implicated in the pathogenesis of various inflammatory and cardiovascular conditions. For instance, high levels of circulating intermediate monocytes are associated with adverse outcomes in coronary artery disease, and S100A8/A9 has been linked to plaque inflammation and instability in atherosclerosis [53]. By drawing these parallels, we propose that expansion of intermediate monocytes and enrichment of S100A8/A9^high^ classical monocytes may contribute to the chronic inflammation and vascular damage seen in RIHD. Definitive testing of myocardial mechanisms will require tissue‐level or in vivo studies.

Another key finding of our study is the bifurcated transcriptional program observed in monocytes after radiotherapy, which underscores the dual nature of the monocyte response. Alongside the inflammatory trajectory culminating in activated classical monocytes, we identified a parallel induction of oxidative stress response genes in earlier‐stage monocytes. This suggests that intermediate monocytes, while primed for inflammatory output, simultaneously activate cytoprotective mechanisms. Radiation is well known to generate reactive oxygen species and cause oxidative stress in exposed tissues. Monocytes are particularly sensitive to oxidative DNA damage, as evidenced by their limited DNA repair capacity, yet they can also adapt by upregulating antioxidant pathways [54]. The enhanced glutathione metabolism and related gene programs we found (Module 2 in our analysis) likely reflect monocytes attempting to counteract radiation‐induced oxidative injury. This adaptation may be crucial for monocyte survival and function: by mitigating DNA damage and maintaining redox balance, intermediate monocytes can continue to proliferate or differentiate into inflammatory cells rather than undergoing apoptosis. Our observations align with the concept of an “adaptive tolerance” or preconditioning in innate immune cells—a phenomenon reminiscent of trained immunity, where a stress exposure leads to epigenetic and metabolic reprogramming that enhances certain functions. In our case, radiotherapy appears to induce a trained‐like state in monocytes, boosting glycolysis and antioxidant defenses (via mTORC1/HIF1*α* and glutathione pathways) to support their sustained inflammatory activity. This complex reprogramming highlights the plasticity of the intermediate monocyte compartment, which can concurrently drive inflammation and guard against stress. However, these transcriptomic signatures are correlative and measured in PBMCs; functional protein secretion and tissue infiltration were not directly assessed in this study.

Relevance of peripheral biomarkers: independent clinical studies have reported dose‐linked changes in circulating cardiac biomarkers (e.g., hs‐cTnT, NT‐proBNP) and early ECG alterations during/after thoracic RT, supporting the concept that early cardiac effects can have peripheral readouts. Moreover, concordance between blood and myocardial single‐cell immune programs has been demonstrated in cardiomyopathy, where paired PBMC and myocardium scRNA‐seq captured disease‐relevant signals; while disease contexts differ, this supports using PBMCs as a systemic window into cardiac‐relevant biology.

Our findings carry several important implications and potential applications. First, intermediate monocytes and their associated molecular signals (such as S100A8/A9, IL‐6, and TNF‐α pathways) could serve as biomarkers for early immune activation in patients undergoing radiotherapy. Monitoring intermediate monocyte levels or inflammatory cytokine profiles in the blood post‐therapy might help identify individuals at higher risk of developing RIHD, enabling closer cardiac surveillance or early intervention. Second, and perhaps most importantly, our study suggests new therapeutic targets to mitigate radiation‐induced inflammation. Additionally, targeting the inflammatory pathways activated in monocytes is a logical strategy. We found IL‐6–JAK–STAT3 signaling to be prominently elevated; IL‐6 is a key driver of acute phase inflammation and is known to contribute to cardiovascular pathology. Monoclonal antibodies against IL‐6 or its receptor (such as tocilizumab) are already in use for inflammatory diseases and have shown the ability to reduce systemic inflammation and even attenuate cardiac injury in clinical settings [55, 56]. Similarly, inhibition of TNF‐α or NF‐κB activation (using existing biologics or small‐molecule inhibitors) might curb the monocyte‐driven inflammatory cascade [57, 58]. Another avenue is to interfere with the alarmin signals like S100A8/A9; experimental inhibitors of S100A8/A9 or its receptor (RAGE/TLR4) have shown promise in reducing inflammation in murine models and could be explored to specifically blunt the proinflammatory output of monocytes after radiation [59, 60]. Beyond direct anti‐inflammatory approaches, boosting the antioxidant capacity of immune cells or providing radioprotective adjuvants might synergize with immune modulation. For example, agents that enhance glutathione or reduce ROS could support the stress‐adaptation program in monocytes, thereby preventing excessive cell death and dysfunctional inflammation [61, 62]. Ultimately, an ideal therapeutic strategy for RIHD might involve a combination of immune modulation and radioprotection—for instance, using cytokine inhibitors or chemokine blockers to control immune activation, alongside antioxidants or DNA repair enhancers to mitigate tissue injury. Importantly, any such interventions would need to be balanced against the primary goal of radiotherapy (tumor control), and thus selective targeting of the pathological immune response (while preserving antitumor immunity) will be key.

In conclusion, our study provides a comprehensive single‐cell perspective on how human immunity—especially the monocyte compartment—responds to thoracic radiotherapy. We uncover a mechanism in which intermediate monocytes assume a central role, undergoing reprogramming that endows them with dual capabilities: propagating inflammation and adapting to oxidative stress. This duality enables them to link radiation exposure to sustained systemic inflammation, providing a basis for the hypothesis that RIHD may be driven not only by direct damage but also by systemic immune remodeling. Our findings bridge an important gap between clinical observations of postradiotherapy inflammation and the cellular and molecular underpinnings of RIHD. By highlighting intermediate monocytes and their bifurcated programs, we open new directions for research and intervention. Future studies should investigate these cells in larger patient cohorts and in animal models of RIHD to validate their causative role. Additionally, exploring therapies that target the monocyte response—from mobilization to inflammatory mediator release—could ultimately lead to strategies that protect the heart and other organs from collateral damage during life‐saving cancer treatments. Addressing radiation‐induced immune reprogramming holds the promise of improving long‐term outcomes for cancer survivors by preventing or ameliorating late‐onset complications like RIHD.

### 4.1. Limitations

This study has several limitations, primarily the small, single‐center cohort (*n* = 6), which may constrain generalizability. Crucially, our analysis was restricted to peripheral blood (PBMCs); while peripheral immune alterations often mirror tissue pathology, we did not analyze myocardial tissue, and thus direct links to cardiac structural changes remain to be confirmed, rendering our insights regarding RIHD pathogenesis hypothesis‐generating rather than definitive. Furthermore, scRNA‐seq provides transcriptomic rather than functional protein data, and pseudotime trajectories are inferential. Therefore, causality cannot be established from this observational study and requires validation in mechanistic animal models or prospective trials.

## 5. Conclusion

In summary, our work reveals that thoracic radiotherapy elicits a dynamic reprogramming of the peripheral immune system characterized by an expansion and activation of intermediate monocytes. These highly plastic monocytes act as a fulcrum of the postradiation immune response, differentiating into proinflammatory effector cells while simultaneously engaging stress‐adaptive mechanisms.

These PBMC‐based observations are hypothesis‐generating regarding the pathogenesis of RIHD. Our findings suggest a model where RIHD may be driven not solely by direct radiation injury to cardiac cells but potentially exacerbated by radiation‐primed circulating immune cells. Translationally, this highlights intermediate monocytes as potential circulating biomarkers for monitoring radiation toxicity. Future mechanistic studies in tissue‐based models are required to definitively establish the causative role of these monocytes in myocardial fibrosis and dysfunction.

## Author Contributions

Jinchen He and Qi Wu conceived and designed the study. Dejun Kong and Yuyuan Wang contributed to patient recruitment, clinical data collection, and ethical documentation. Tianqi Wu and Hong Yang conducted experimental operations and assisted with manuscript preparation. Chengwei Zhang and Qiuhong Chen performed statistical analysis and provided methodological support for data interpretation. Jinchen He drafted the manuscript. Qi Wu supervised the entire project and revised the manuscript critically for intellectual content.

All authors reviewed and approved the final manuscript.

## Funding

This research was supported by the following funding bodies: Sichuan Provincial Department of Science and Technology, Sichuan Science and Technology Innovation “Seedling Project” (Grant No. MZGC20240008); China National Nuclear Corporation, CNNC Medical “Nuclear Medicine Technology Innovation” Program (Grant No. ZHYLZD2025015); Chengdu Municipal Health Commission, 2025 Medical Research Project of Chengdu (Grant No. 2025069); Sichuan Provincial Key Laboratory of Irradiation Preservation and Effect, General Project (Grant No. 2025FZBCY09); and Sichuan Provincial Department of Science and Technology, 2024 Central Government Guides Local Science and Technology Development Fund (Grant No. 2024ZYD0091).

## Disclosure

The funders had no role in study design, data collection and analysis, decision to publish, or preparation of the manuscript.

## Ethics Statement

All procedures performed in this study involving human participants were in accordance with the ethical standards of the institutional research committee and with the 1964 Helsinki Declaration and its later amendments. The study protocol was approved by the Ethics Committee of The Second Affiliated Hospital of Chengdu Medical College (approval number: YJ‐2024‐051). All participants provided written informed consent prior to inclusion.

## Conflicts of Interest

The authors declare that the research was conducted in the absence of any commercial or financial relationships that could be construed as potential conflicts of interest.

## Supporting Information

Supporting information for this article includes a document containing two supporting figures.

Supporting Figure S1, Cell‐type‐specific metabolic rewiring after radiotherapy revealed by scMetabolism analysis.

Supporting Figure S2, Global and subtype‐specific CytoTRACE variation before and after radiotherapy.

## Supporting information


**Supporting Information** Additional supporting information can be found online in the Supporting Information section.

## Data Availability

The data that support the findings of this study are available from the corresponding author upon reasonable request.
