# Supplementary material for: Radiotherapy Reprograms Intermediate Monocytes Into Proinflammatory Drivers of Systemic Inflammation in Radiation‐Induced Heart Disease
Source: Cardiol Res Pract. 2026 Apr 17;2026:1117746. doi: 10.1155/crp/1117746 (PMC13090681; doi:10.1155/crp/1117746)
Supplement: Supplementary file 1 — Supporting Information Additional supporting information can be found online in the Supporting Information section. [file CRP-2026-1117746-s001.docx]

****
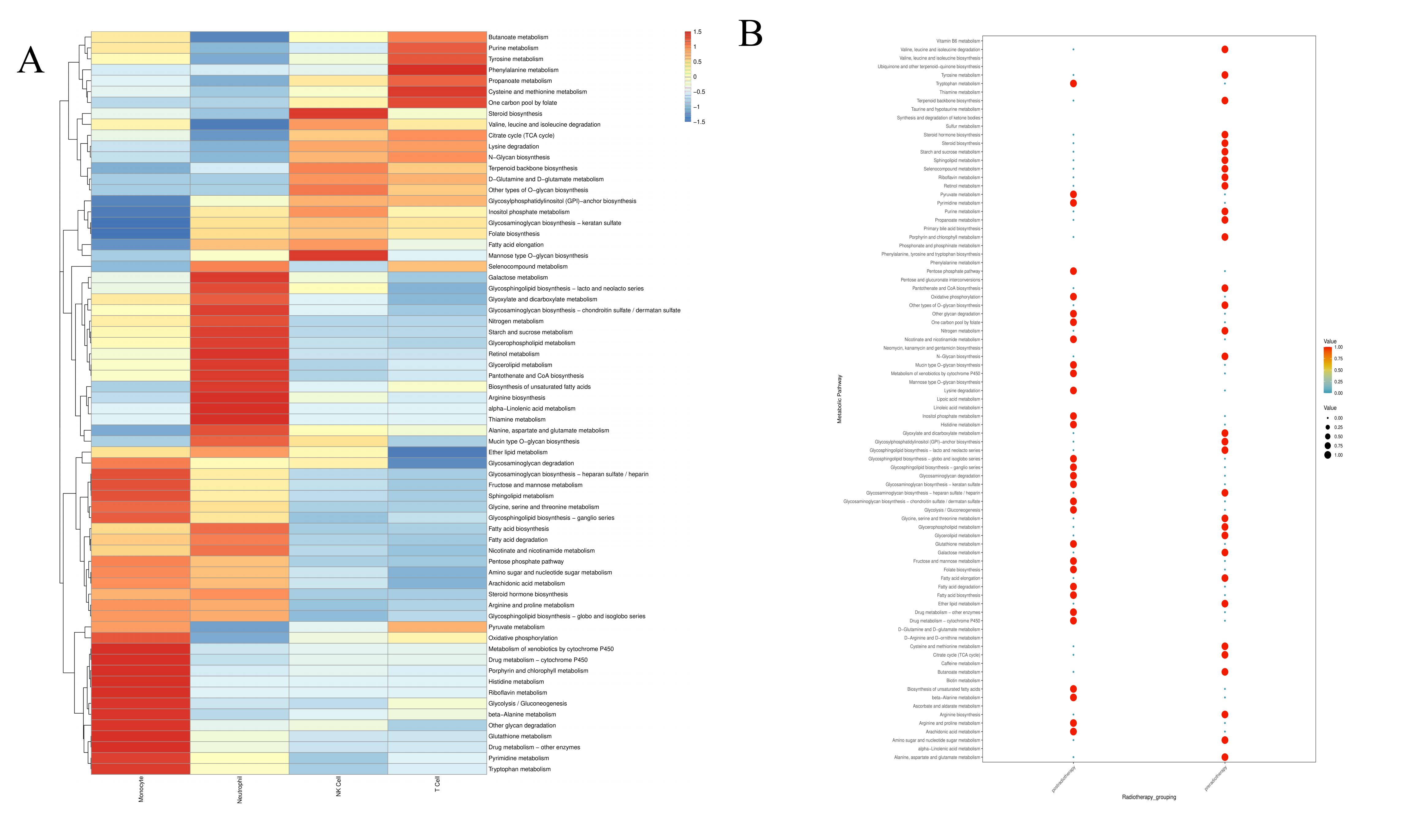
****

****Supplementary Figure S1. Cell-type-specific metabolic rewiring after radiotherapy revealed by scMetabolism analysis.****

**(A)** Row-scaled heatmap of KEGG pathway activity scores inferred from scMetabolism across four major immune cell types, highlighting prominent upregulation of glycolysis, glutathione metabolism, and fatty acid degradation in monocytes.
**(B)** Monocyte-specific heatmap showing enhanced metabolic pathway activity after radiotherapy, supporting the transcriptionally predicted metabolic reprogramming observed in GSVA (Figure 3F–H).


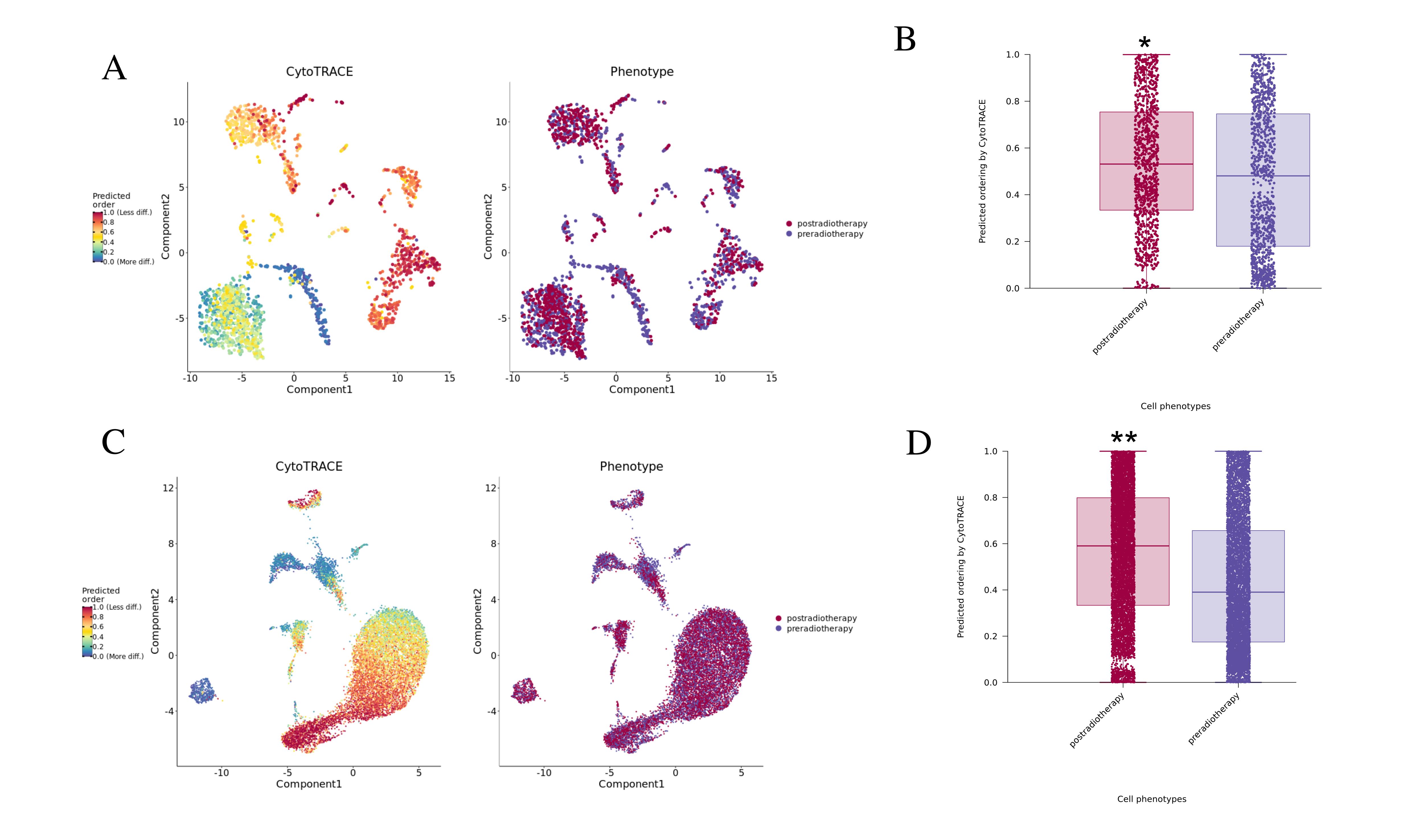


****Supplementary Figure S2. Global and subtype-specific CytoTRACE variation before and after radiotherapy.****

(A) UMAP visualization of peripheral immune cells colored by CytoTRACE scores (left) and by treatment group (right). CytoTRACE scores represent inferred differentiation status, where higher scores (red) indicate less differentiated and more transcriptionally plastic states. Cells from post-radiotherapy samples (red dots) are more enriched in regions with higher CytoTRACE scores, suggesting a shift toward a more progenitor-like immune landscape following radiotherapy.

(B) Boxplot comparing CytoTRACE scores between pre-radiotherapy (purple) and post-radiotherapy (pink) immune cells. Post-radiotherapy cells exhibit a higher median CytoTRACE score, indicating reduced differentiation and increased plasticity across the immune compartment. Statistical

(C) UMAP plot of classical, intermediate, and non-classical monocyte subtypes colored by CytoTRACE score (left) and by treatment group (right). Post-radiotherapy monocytes (red) are enriched in less differentiated zones.

(D) Boxplot comparing CytoTRACE scores between pre- and post-radiotherapy Of classical, intermediate, and non-classical monocyte subtypes. A significant increase in CytoTRACE values is observed in post-radiotherapy cells, confirming that radiation promotes a shift toward earlier differentiation states within the monocyte compartment.

significance was calculated using the Wilcoxon rank-sum test, with pre-radiotherapy used as the reference group**.** *p < 0.05, **p < 0.01, ***p < 0.001, ****p < 0.0001
